# Utility of social networks and online data collection in nursing research: Analysis of Spanish nurses’ level of knowledge about palliative care

**DOI:** 10.1371/journal.pone.0197377

**Published:** 2018-05-14

**Authors:** Elena Chover-Sierra, Antonio Martínez-Sabater

**Affiliations:** 1 Nursing Department, University of Valencia, Valencia, Spain; 2 Hospital General Universitario, Valencia, Spain; University Antwerp, BELGIUM

## Abstract

**Introduction:**

Internet-based social networks are used by nurses with different purposes, including the creation of working groups and to share and create knowledge.

**Purpose:**

To evaluate the utility of social networks in the dissemination of an online questionnaire and to measure Spanish RNs’ knowledge about palliative care.

**Methods:**

A descriptive cross-sectional study was carried out. Using social networks we distributed an online questionnaire with the Spanish version of Palliative Care Quiz for Nurses (PCQN-SV) throughout August 2015.

**Results:**

A direct relationship between the number of responses and the questionnaire’s apparitions in each social network was found. Comparing the results obtained by the 446 RNs participating in this study with those obtained in the study to pilot the PCQN-SV we identify that differences found are related to the participants’ features (years of experience and hours of training in palliative care) and not to the type of questionnaire they answered.

**Conclusions:**

Social networks have shown to be a useful tool for nursing research by its ability, to recruit participants as well as to collect data, so their role as an instrument of research should be considered.

## Introduction

When we talk about social networks we refer to a set of Internet-based applications that allows the connection and simultaneous interaction of different individuals, with some common interest or objectives, although they are in very distant geographical environments; this connection is established with very little effort of the netizens, these social networks help also to share information, in the form of written texts or in other formats such as image, audio, video … [1, 2]

These social networks have had a major expansion in recent years, to become channels of communication with potential scope and impact and a capacity of greater interaction and dynamism than any other media [3, 4]. Their use has increased among health professionals, since the possibility of contact with other professionals and develop networks to generate and disseminate knowledge has been found, among other multiple utilities. These networks serve, at the same time, as a repository of information, which increases exponentially the possibilities of teamworking and to track in real time the generated contents, aspects that healthcare professionals must take into consideration when using them, to achieve greater profitability and disseminate information in the most efficient way possible [5–7].

In fact, in the majority of studies in which they analize the use of networks by healthcare professionals it is highlighted the interest in analyzing the pace of dissemination of information on events such as scientific meetings and the content analysis of the disseminated information [8, 9], instead of their use as useful tools in research, and especially in small scale research [10, 11].

Among social networks with greater frequency of use by both the general population and healthcare professionals we can find Facebook and Twitter, two platforms with different features, having been used previously in different research works in the health sector, in which it was intended to analyze the diffusion speed of some content, as a mean to disseminate the information generated in scientific meetings, their helpfulness to create networks or their opportunities to recruit participants in research, among other aspects [12–14].

Facebook is a social network in which pieces of information and contents are shared with other users from the profile created by the user (these contents are shared publicly or only with selected followers); this network also allows the creation of groups/communities of subjects who share certain content [15]. On the other hand, Twitter is a social network for microblogging type, in which users communicate instantly via short messages of up to 280 characters (tweets), whose dissemination speed will depend on the number of followers any user has and the number of times that a message is shared (retweeted). In this network, the information is shared with all those who compose it, although you can restrict access to tweets that an individual publishes [10, 16–18].

It is well known the difficulty of accessing the target population while conducting a research paper, and the time that sometimes is needed to get to a volume of answers suitable for obtaining results that can be transferred to other populations; hence, the possible use of any of these networks to recruit participants in a short period of time has already been studied in some researches and its effectiveness to achieve this objective has been analyzed [19].

From several associations working in the field of palliative care it is postulated that any professional nurse should acquire a level of basic knowledge in this area, therefore, this tuition should be given during their undergraduate training [20–23]. Different studies have analyzed the situation of nurses’ training in palliative care in many countries and have developed proposals to improve this training, as it is the case of the proposals elaborated by the EAPC, including the training program European Certificate in Essential Palliative Care (ECEPC), offered to professionals working in the field of palliative care, specially in hospices, was stablished [24, 25] and programs such as End-of-Life Nursing Education Consortium Training Program ELNEC, developed in the United States and then spread to Europe and Asia [26–28].

However, in Spain, and in spite of the recommendations for the training of nurses in this area [20, 29], we still offer an heterogeneous training to our university students and not all the nurses have even this basic formation [30].

This work aims for analyzing the utility as a mean for sharing a questionnaire to determine the level of knowledge in palliative care among Spanish nurses of the two most used social networks in our environment, Facebook and Twitter, taking into account their distinguishable features, which will determine the diffusion speed of the contents in each of them, and to define the possibility of using them within a research project which aims to study the level of knowledge in palliative care of nursing professionals throughout our country.

To evaluate nurses’ knowledge about palliative care, the Spanish version of PCQN has been chosen. The PCQN is a self-administered questionnaire on basic knowledge for nursing in the field of palliative care, which includes questions on three aspects of the palliative care: philosophy and principles, psychosocial aspects of palliative care, and pain and other symptoms’ control; it consists of 20 true/false/don’t know questions, and was originally designed at the University of Ottawa, translated into different languages and used in several research studies around the world [31–35]. The Spanish version of the PCQN (PCQN-SV), piloted in a group of 159 registered nurses who worked in a Spanish hospital, presents appropriate content validity indexes (CVI-S = 0.83) and internal consistency (KR-20 = 0.72, Cronbach’s alpha = 0.67) [36, 37]

After the completion of this pilot study, the amendments proposed to this version were included, and we developed a modified version of the PCQN-SV, the one that has been used in this study, in order to go on with its validation process.

Our intended purpose is mainly to confirm that the use of online questionnaires and collecting information through the Internet and social networks does not affect the obtained results, and therefore, that we have a useful and accessible tool that allows us to make profit and improve nursing research processes.

## Purposes

- Assess the use of social networks for the data collection in research studies.
- Determine the level of knowledge in palliative care of the participating nurses.
- Identify differences in the results according to the method used to disseminate the questionnaire (traditional versus social network dissemination)

## Material and methods

### Design

A descriptive cross-sectional study was designed, and then developed during the month of August 2015.

### Study population and participants’ selection

The study population were Spanish registered nurses, users of social networks who accepted to participate in the study, since we expected not only to analyze the dissemination of the questionnaire but also to continue with the validation process of the Spanish version of the PCQN (PCQN-SV)

Initially, the link that allowed accessing to the questionnaire was shared on Twitter accompanied by a presentation message that included #enfermeria (#nursing) and #paidadospaliativos (#palliativecare) as hashtags, in order to draw the attention of nursing professional users of that network. In the case of Facebook, the questionnaire was shared from the personal profiles of researchers on the walls of certain groups related to nursing, composed by nurses who use that network.

From this initial spreading a snowball sampling was set out, in which the participants themselves were responsible for the dissemination and distribution of the questionnaire from their own profiles in social networks if they considered it appropriate.

### Data collection instrument

We designed a data collection instrument by means of Google Formular, which allows its distribution as a link to the website where it is hosted and, therefore, its dissemination via the internet, and the possibility to reply to it not only from a computer, but also from any other electronic device with internet access; with this instrument we collected information about the study population’s features and also the responses to the Spanish version of the questionnaire PCQN.

To analyze the diffusion of the questionnaire, the statistics of Facebook and Twitter were reviewed in order to manually count the number of times that each message was shared in each social network.

We considered only the first month of questionnaire diffusion using social networks, to compare these results with those of the study to pilot the PCQN-SV, a study developed during one month among registered nurses working in a Spanish hospital.

### Data analysis strategy

Non-parametric correlation techniques were used to analyze the relationship between the times that the questionnaire was shared on each social network and the number of questionnaire’s answers.

Also, a descriptive univariate analysis of the results of the questionnaire (percentage of right and wrong answers for each item and also the overall percentages) and of the variables used to characterize the study population was performed, as well as descriptive bivariate analysis, through correlations’ study and test of independence between variables concerning population’s features and the results obtained on the questionnaire, using non-parametric test.

Correlations between results obtained in the different subscales and the global questionnaire were also analyzed. In order to identify whether these relationships were due to the participants’ features or influenced by the type of study developed, we adjusted these correlations for the two variables whose differences shown to be statistically significant between both groups.

Analyses were performed with the statistical software SPSS v.21 and in all cases was established as statistically significant a p-value < 0.05.

Following the guidelines of the item response theory, difficulty and discrimination indexes have also been calculated for each item composing the questionnaire, on the basis of the right answers obtained for the study population.

### Ethical aspects

The process of distribution of the questionnaire allows that only the subjects who want to participate will answer or share it, also the form, in its first page before any questions, makes reference to the objectives of the study and the anonymity and confidentiality of data collected. Then it explicitly indicates to the network users that they must agree to participate in order to access the complete questionnaire, so that, only those who selected the option “yes” when it was stated “do you accept to answer these questions?”, giving their informed consent, would participate in the study.

Any google Formular is associated to a gmail account, so to create this type of formular in order to collect some information an individual should comply the terms of use of gmail and their related products. The researchers have also complied the terms of use of both social networks used in the dissemination of questionnaire.

## Results

### Spreading pace of questionnaire on social networks

This diffusion was analyzed during the first 30 days, considering the rate of dissemination of the questionnaire as well as the number of times shared daily in each social network and analyzing its relationship with the number of answers obtained.

**Fig 1:**
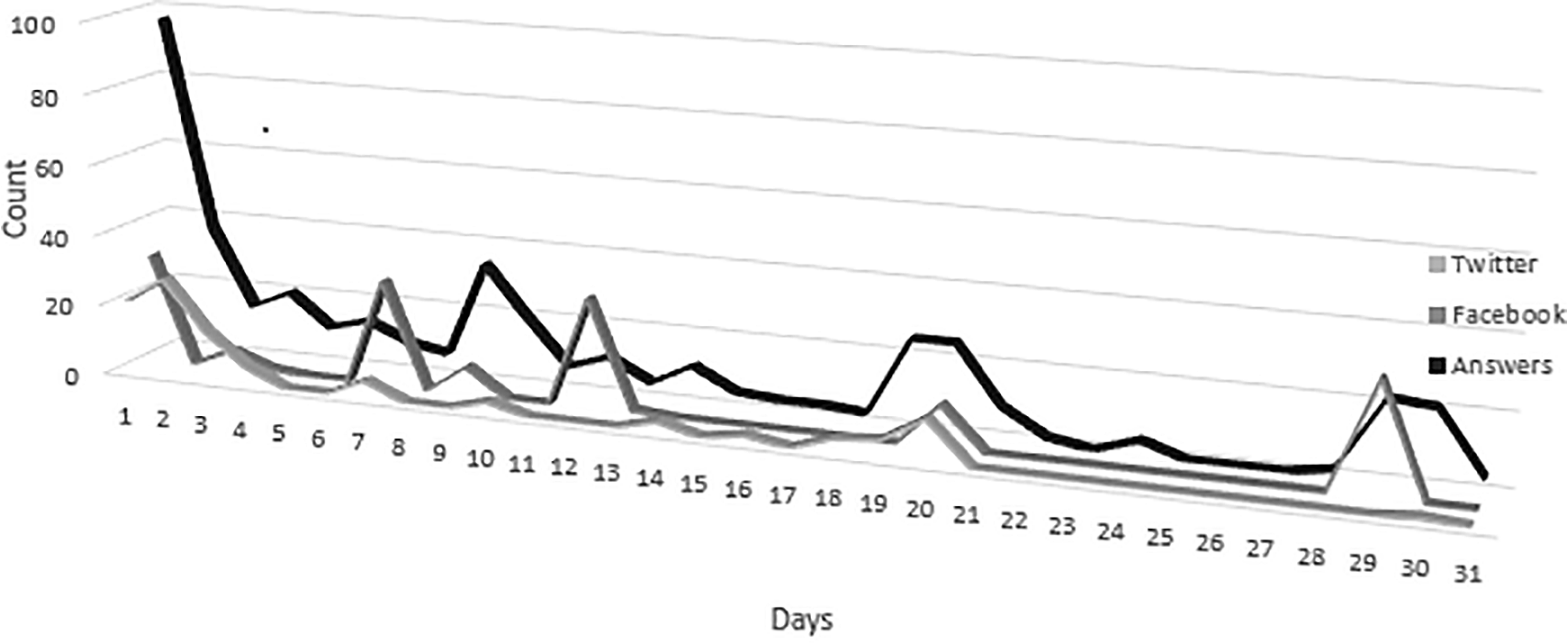
Relationship between the number of responses to the questionnaire and its rate of diffusion in social networks.

We found a statistically significant relationship between the number of answers and the number of times that the publication was shared in each social network (rho = 0. 52, p < 0.001 in the case of Facebook and rho = 0. 58, p < 0.001 in the case of Twitter). This relationship is represented in Fig 1.

### Features of study participants

446 Spanish registered nurses working in different environments, mainly in acute tertiary hospitals (45%) and primary care (22.5%) took part in this study.

Table 1 shows the sociodemographic characteristics and aspects relating to the level of training and experience in palliative care referred by the participants. 39.01% of them indicated having both experience and training in this field, and 18.06% indicated not having any experience or training.

**Table 1:** Features of studied population (n = 446) and comparison with pilot study (n = 159).

	Current study			Pilot study			p-value
	Average±SD	Range	n(%)	Average±SD	Range	n(%)	
***Age***	38.30± 1.90	21–66		39,51±10,25	21–62		0.21*
***Gender***							0.43^**£**^
**Female**			355(79.6)			134(84.28)	
**Male**			91(20.4)			25(15.72)	0.14*
***Professional experience (years)***	14.12±10.85	0–43		13.96±10.79			
***Academic qualification***							**<0.01**^**£**^
**Basic nursing degree**			312(69.9)			136(85.5)	
**Master degree**			108(24.2)			23(14.5)	
**PhD**			11(2.5)			0	
**Nurse specialist**			15(3.4)			0	
***Experience in PC***							**<0.01**^**£**^
**YES**			232(52)			87(54.7)	
**NO**			214(48)			72(45.3)	
***Experience in PC†*** ***(years)***	2.21±4.88	0–43		2.84±3.05	0–24		<0.05*
***Training in PC†***							0.33^**£**^
**YES**			305(68.4)			102(64.2)	
**NO**			141(31.6)			57(35.8)	
***Training in PC†*** ***(Type)***							0.45^**£**^
**University education**			103(27.6)			32(31.4)	
**Ongoing training**			141(37.8)			30(29.4)	
**University + Ongoing**			42(11.3)				
**Postgraduate courses**			21(5.6)			6(5.9)	
**Postgraduate + ongoing training**			16(4.3)			5(4.9)	
**Master degree**			13(3.5)				
**Master+ ongoing training**			2(0.5)				
**Postgraduate+ Master+ongoing tr.**			5(1.3)				
**University +Postgraduate**			5(1.3)				
**University+Postgrad+ Ongoing**			9(2.4)				
**University+ Master degree**			2(0.6)				
**Others**			14(3.8)			5(4.9)	
***Training in PC†*** ***(Hours)***	101.10±395.99	0–7200		94.86±65.79	0–500		<0.01*
***Training in PC†*** ***(Hours)***							**<0.01**^**£**^
**< 20**			93(30.5)			30(29.4)	
**20–50**			73(23.9)			33(32.4)	
**50–100**			57(18.7)			28(27.5)	
**> 100**			82(26.9)			11(10.8)	

† PC. Palliative care

* Mann Whitney test

^£^ Chi-square test

### Nurses’ level of knowledge in palliative care

When calculating the percentage of right and wrong answers to each question comprising the questionnaire we found a great variability in these figures, being questions 4, 8, 1 and 18 those with highest percentage of right answers and questions 5, 20, 19 and 17 which obtained greater percentage of errors.

If we look at our results as a whole, we find a global percentage of right answers of 59.38% (I.C. 58.13–60.67; SD = 13.52) and a global percentage of wrong answers of 29.57% (I.C. 28.36–30.77; SD = 12.61)

Table 2 shows the results obtained for each of the three subscales of the questionnaire.

**Table 2:** Results for each subscale of PCQN-SV.

	RA^†^ (%)		WA^‡^ (%)	
	Average± SD	C.I	Average± SD	C.I
**Philosophy and principles**	65.30±24.17	62.78–67.60	27.52±23.77	25.28–29.99
**Psychosocial aspects**	38.94±29.66	36.25–41.70	47.31±27.85	44.77–50
**Symptoms’ control**	62.28±15.50	60.87–63.71	26.11±13.53	24.87–27.30

† RA: Right answers

‡ WA: Wrong answers

### Variables that influence the level of knowledge about palliative care

We found differences in the global results of the questionnaire, always in favor of individuals with experience or training in the field of palliative care. Participants with experience in palliative care had a percentage of right answers of 62.65% (C.I. 60.88–64.42; SD = 13.67, while participants with training in this area had a percentage of right answers of 60.97% (C.I. 59.46–62.47; SD = 13.33); although these differences are only statistically significant for the percentage of correct answers (p<0.001 for both groups).

The results obtained in each subscale, with differences among participants are shown in Table 3.

**Table 3:** Results for each subscale of the PCQN-SV according to the experience and training in palliative care of the participants.

			RA^†^ (%)	p-value^§^	WA^‡^ (%)	p-value^§^
			Mean±SD		Mean±SD	
**Experience in palliative care**	**YES**	**Philosophy and principles**	66.27**±**24.88	p = 0.35	28.77**±**23.93	p = 0.22
	**NO**		64.25**±**23.39		26.17**±**23.58	
	**YES**	**Psychosocial aspects**	43.39**±**29.66	**p < 0.001**	46.84**±**27.91	p = 0.62
	**NO**		34.11**±**28.97		47.82**±**27.84	
	**YES**	**Symptoms’ control**	65.98**±**15.75	**p < 0.001**	25.07**±**13.96	p = 0.09
	**NO**		58.27**±**14.20		27.25**±**12.99	
**Training in PC**	**YES**	**Philosophy and principles**	66.31**±**24.38	p = 0.16	27.29**±**24.22	p = 0.61
	**NO**		63.12**±**23.64		28.01**±**22.84	
	**YES**	**Psychosocial aspects**	39.12**±**30.09	p = 0.81	47.76**±**28.41	p = 0.64
	**NO**		38.53**±**28.81		46.33**±**26.66	
	**YES**	**Symptoms’ control**	64.36**±**15.21	**p < 0.001**	25.44**±**13.23	p = 0.18
	**NO**		57.77**±**15.21		27.55**±**14.10	

† RA: Right answers

‡ WA: Wrong answers

^**§**^ Mann-Whitney test

Whether the effect of these two variables is combined, we could see that participants with experience and training got better results on the questionnaire, with a percentage of right answers of 59.24% (C.I. 57.49–60.99; SD = 14.02) and a percentage of wrong answers of 29.96% (C.I. 28.34–31.58; SD = 12.99), while participants without experience or training obtain a 53.72% of right answers (I.C. 67.57–80. 67; SD = 31. 22) and a percentage of wrong answers of 59.24 (I.C. 51.20–56.23; SD = 13.97), being these differences statistically significant only for the percentage of right answers (p < 0.01).

We did not find statistically significant differences according to other population’s features such as the area in which they develope their job, although the best results on the questionnaire were achieved by professionals working in palliative care services, with a 70% of right answers (C.I. 67.57–80.67; (SD = 31.22), and those working in at-home hospitalization, with a 67.50% (C.I. 61.33–72.33; (SD = 17.68). On the other hand, the participants working in public health institutions obtained the higher percentage of errors, 39% (C.I. 24.84–53.16; SD = 11.40) followed by those working in nursing homes, with 31.58% (C.I. 27.31%-35.85%; SD = 13)

We also studied the correlation between years of professional experience in the field of palliative care and hours of training in this area and the results obtained by the participants both in the global questionnaire and in each subscale.

Regarding the years of experience, we found statistically significant relationships with the percentage of right answers in the whole PCQN (rho = 0.23, p < 0.001), and in the subscales of psychological aspects (rho = 0.15, p < 0.05) and symptom´s control (rho = 0.23, p < 0.001). In the case of the hours of training, we only found statistically significant relationships with the percentage of right answers in the global questionnaire (rho = 0.20, p < 0.001) and in the scale of symptom’s control (rho = 0.24, p < 0.001).

Correlations between results obtained in the three subscales are shown in Table 4.

**Table 4:** Correlation between the results obtained in the different subscales of PCQN-SV.

			Philosophy and principles		Psychosocial aspects		Symptoms’ control	
			RA^†^	WA ^‡^	RA^†^	WA ^‡^	RA^†^	WA ^‡^
**Philosophy and principles**	**RA**^**†**^	**rho**	—					
		**p**						
	**WA** ^**‡**^	**rho**	-0.78	—				
		**p**	**<0.001**					
**Psychosocial aspects**	**RA**^**†**^	**rho**	0.19	-0.16	—			
		**p**	**<0.001**	**<0.01**				
	**WA** ^**‡**^	**rho**	-0.14	0.23	-0.70	—		
		**p**	**<0.01**	**<0.001**	**<0.001**			
**Symptoms’ control**	**RA**^**†**^	**rho**	0.18	-0.21	0.14	-0.03	—	
		**p**	**<0.001**	0.65	**<0.01**	0.49		
	**WA** ^**‡**^	**rho**	-0.14	0.21	-0.11	0.19	-0.59	—
		**p**	**<0.01**	**<0.001**	**<0.05**	**<0.001**	**<0.001**	

† RA: Right answers

‡ WA: Wrong answers

### Comparison of results with PCQN-SV pilot study

Firstly, we studied differences between both populations, which could explain their outcomes; in Table 1 we have shown the caracteristics of both populations and its main differences.

Regarding the differences in the results obtained in both studies, we could say that the participants in pilot study obtained worse results than the participants in this work; i.e., lower percentage of right answers and highest percentage of wrong answers, both in the overall results whilst for each subscale, as shown in Table 5.

**Table 5:** Differences of results in PCQN-SV between current study and pilot study.

		RA^†^ (%)	p-value^§^	WA^‡^ (%)	p-value^§^
		Mean±SD		Mean±SD	
**Global PCQN**	**Current study**	59.38**±**13.52	**p < 0.001**	29.57**±1**2.61	**p < 0.01**
	**Pilot study**	54.02**±**13.38		33.36**±1**2.04	
**Philosophy and principles**	**Current study**	65.30**±**24.17	**p < 0.001**	27.52**±**23.77	**p < 0.001**
	**Pilot study**	55.82**±**24.71		36.32**±**23.82	
**Psychosocial aspects**	**Current study**	38.94**±**29.67	p = 0.45	47.31**±**27.85	p = 0.09
	**Pilot study**	31.45**±**28.13		54.93**±**27.08	
**Symptoms’ control**	**Current study**	58.73**±**16.68	**p < 0.05**	26.11**±**13.53	p = 0.58
	**Pilot study**	62.28**±**15.50		27.43**±**15.35	

† RA: Right answers

‡ WA: Wrong answers

^**§**^ Mann-Whitney test

When correlations between numeric variables were adjusted in both groups by the years of experience and the number of hours of training in palliative care, the results were very similar as those of non-adjusted correlation. This finding tells us that the characteristics of the participants and not the difference in the methodology of the study, are those which justify the different results obtained in each group, and that there are no differences related to the methodology used for the dissemination of the questionnaire and data collection.

### Analysis of reliability and difficulty of PCQN-SV

When we study the reliability of the PCQN-SV after its use in this population we find a KR-20 of 0.74 and a Cronbach’s alpha of 0.69.

These indexes are slightly superior to those obtained in the pilot study (KR-20 0.72, Cronbach’s alpha 0.67), as was expected, since modifications in the questionnaire have been made after its completion with the intention of improving its reliability, although we see that they are quite stable, which is also determined by the fact that both studies have been developed in populations with similar characteristics.

When we study the percentage of right and wrong answers for each one of the questions that compose the questionnaire, we can appreciate that there are 7 items with a percentage of right answers under 50% and 10 items with over 25% of wrong answers.

The average difficulty index of the questionnaire is 0.59. Three items have a difficulty index over 0.60 (3,9,12) and five items even over 0.80 (1,4,8,15,18), which let us classify them as easy or very easy; and five others (6, 14, 16, 17, 19) can be considered as difficult or very difficult, with difficulty indexes between 0.20 and 0.45.

According to their discrimination indexes, we can define eight items as very good ones (with discrimination indexes higher than 0.40), five items (with indexes between 0.3 and 0.39) as good, four items as fair (indexes between 0.2 and 0.29) and three as bad (with lower or negative discrimination indexes)

## Discussion

In this study, we have found that the use of social networks in research allows us to reach a significant number of participants in a short period of time, if we compare it with the usual recruitment method. In this sense, we can compare the number of questionnaires obtained during 30 days with this system, 446 questionnaires against 159 obtained in the pilot of the Spanish version of the PCQN, in which it was distributed among professionals in a single hospital, through the usual procedure of hand-to-hand delivering in the different units [37].

It is important to note that these 446 questionnaires collected in 30 days have been obtained from nurses all around the Spanish territory, something that hardly could be made through the usual procedure, without getting in contact and collaborating with researchers from different health services that become responsible for the distribution and collection of questionnaires.

Many works that intend to guide health professionals in the management of social networks from a professional point of view and not only on a personal one are appearing continuously, some of them even referring to their potential uses in research studies [38, 39].

A review [10] published in 2015 analyses 31 research projects in the field of health sciences in which Twitter was used. It indicates that both data collection and analysis used to be a long and expensive process, and that the use of this platform for these processes could make them more economical and less laborious. We have also verify it with our work, since the possibility of distributing the data collection instrument through social networks has allowed us to reach a greater number of subjects than in the pilot study. This capability to reach to a higher number of participants is also shown in two studies [19, 40] which talk about the utility of the social network Facebook to get larger numbers of participants for their studies rather than the conventional methods of recruitment, during similar time periods. Moreover, this way of recruitment is more economical, something that we could also assess, since it was not necessary to invest any money neither for the questionnaire dissemination nor for data collection in the current work.

Thus, social networks would be a very useful tool when it comes to developing a snowball sampling, since we are moving into a niche of population with the characteristics we are looking for, and the participants in the study themselves are who continue with the broadcast, making it possible to get every time a greater number of participants, with similar characteristics, to increase the sample size up to the required.

If we compare this system of dissemination of questionnaires and collection of information with the habitual one based on delivering questionnaires on physical support and subsequent manual introduction of the collected data to a traditional database, we can see that it offers advantages in terms of time reduction, something that will expedite the following phases of the research process.

However, we must bear in mind that with this mode of questionnaires’ distribution we are reaching only a small percentage of the whole nurses’ population, in this case those who have a presence in the analyzed social networks. It is also possible that those interested in the issue of palliative care show more interest in participating in our study, since the hashtag #palliativecare (#cuidadospaliativos) appeared in the tweet containing our questionnaire. Even so, in the studied subjects we have not found a greater percentage of subjects with experience in palliative care, although they related more training in this area.

Obviously, this dissemination with the help of social networks can make us lose control over the process, since we cannot ensure that people who answer the questionnaire are really registered nurses (something perhaps easier when distributing it in the health center’s physical space). So we try to control the process, including hashtags in the message that accompanied the questionnaire in Twitter, and also posting messages in Facebook groups’ walls whose theme was related to nursing.

When studying the relation between the number of publications of the questionnaire in each of the social networks and the number of responses, we observe that it seems to be a little greater influence of Twitter, since the number of interactions on this social network has been higher, and also the value of Pearson's correlation coefficient is slightly greater. Nevertheless, we shouldn't forget that the characteristics of both networks would also have influence on the messages’ rate of dissemination, and the time of permanence of the messages in each one of them.

Only three questionnaires have been discarded at the moment of the statistical analysis, those whose respondents indicated not to be Spanish, variable that was included in the questionnaire to be sure to include only Spanish nurses, because we wanted to validate the version in Spanish for our country and we thought that some concept could be interpreted somewhat differently by professionals from other Spanish-speaking countries.

We designed the online form so that all of its questions were of mandatory response. It was done in order to try to reduce to the maximum the questionnaires to be eliminated due to “deficiencies” when they were answered, as it came up with some questionnaires delivered on paper in the pilot study, eliminated because participants did not answer all the questions of the PCQN-SV_._

The overall results of this study, with a percentage of correct answers in PCQN slightly inferior to 60%, are better than those obtained in pilot study to validate the Spanish version of the PCQN, in a study performed in Florida with nurses working in pediatric area [41] and in the validation study of the French version of the PCQN [33]. On the other hand, our results in the overall PCQN are worse than those obtained in other studies, with results superior to 60% of correct answers, in which participating nurses developed their professional activity in different work environments and in different countries such as Korea, Canada, Australia, Ireland and the USA [42–48].

PCQN has also been used to evaluate nurses’ level of knowledge in palliative care and in countries where it has very little development. In a study carried out in four hospitals in Addis Ababa [49], they found that only 30% of the participants had a good level of knowledge in the field of palliative care. In Saudi Arabia, in a study conducted in a specialized palliative care hospital, participants obtained a percentage of right answers of 45,30% in PCQN [31], and in Jordan, nurses working in five hospitals got 41.5% of right answers in the questionnaire [50]; although in these last two studies, their authors also considered a limitation the fact of having used the English version of the PCQN and not a version in Arabic, which would have avoided difficulties of understanding among participants.

If we consider the results obtained by participants in the current study according to their levels of experience and training palliative care, we find that both participants with experience and with training in palliative care got better results on the questionnaire. These results are similar to studies in which professionals with previous training or participating in training programs in the field of palliative care [31, 44, 45, 48], and also those who work in environments such as long-stay centres, nursing homes, oncology units and services of palliative care [42, 43, 46, 47] obtained the best results. Furthermore, in the studies developed in Ethiopia, Jordan, and Saudi Arabia the lack of training in palliative care is considered as one of the main factors that would explain the deficit of knowledge of nurses in this area [31, 49].

In this work, the highest percentage of right answers was achieved in the scale “philosophy and principles of palliative care”, while the subscale “psychosocial aspects” obtained the worst percentage of right answers. In the PCQN-SV pilot study [37] we found, like Brazil [43], that the greater percentage of right answers was obtained in the subscale “symptoms’ control”. Results of both studies were, nevertheless, lower than those obtained in the actual study, in which the participants have obtained higher percentages of right answers in the three subescales. Al Qadire [50] analyzed also the results for each of the subscales, finding that it was the scale of “symptoms’ control” the one in which the participants obtained the best results, while the scale of psychosocial aspects obtained the best results in the studies performed by Raudonis and Ronaldson, among nurses who worked in nursing homes [47, 51].

We have found differences between the results of participants in current study and those from the pilot study performed to validate the PCQN-SV. Participants in this study obtained higher percentages of right answers, which could be explained by the fact that the participants in this study have more training hours in the field of palliative care, and it is also a more recent formation. Nevertheless, these differences are not statistically significant in the subscale psychosocial aspects of palliative care, an aspect that should be introduced in training programs, nowadays excessively focused on aspects as the control of symptoms.

The Spanish version of PCQN has shown adequate levels of internal consistency, in the populations in which has been studied, although in this actual work, and after the changes made after the validation study, we found slightly higher values of the Cronbach’s alpha, and the coefficient KR-20, as we expected.

In the difficulty study, we obtained values very similar to those of the pilot study, with 30% of the items considered as easy or very easy and another quarter considered as difficult or very difficult; in this way, we could say that it is a fairly balanced questionnaire regarding its level of difficulty, and that these features remain when it is used in a population that is slightly different from the one used for the pilot study.

If we also analyze the discrimination indexes we can say we have a questionnaire that is useful to study the level of knowledge in the field of palliative care among Spanish nurses, because more than a half of the questionnaire items are answered correctly by people with higher level of knowledge and there are a few items that should be reformulated.

PCQN-SV allows us to analyze the results of each item individually and, therefore, identify those questions in which the studied population obtained worse results, lower percentage of correct answers or greater percentage of wrong answers, and thus to adjust the content of training programs in palliative care according to the lacks in knowledge shown by this population, on the basis of those wrong ideas /deficits shown by participants.

The differences found in the study of the internal consistency and difficulty of the questionnaire could be due to both the modifications made to the questionnaire after their piloting and elaboration of the final version used in this and subsequent works and the characteristics of the population participating in this study, with a more recent formation and more hours of training in the field of palliative care.

### Limitations and strengths

This study presents a series of limitations related to the methodology used for the dissemination of the questionnaire.

The fact of having a questionnaire disseminated through social networks means that any participant must have an active account in any of the two networks, during the period of the work, so the representativeness of the sample is reduced and it makes it difficult to extrapolate the results to the population of Spanish nursing professionals.

Another limitation, related to our poor management of these networks, may have generated distortion in the analysis of the questionnaire dissemination, since it was only performed a manual count of the number of times that the message was shared in each network, without using any software as one of those used, for example in Twitter, to measure the number of impressions generated by a tweet (and not only the number of times it was shared)

Even so, we consider that these limitations do not affect our objectives, since at no time we have tried to establish the level of knowledge in palliative care of all Spanish nursing professionals, but only among the participants in this study, since our main intention has been the validation of both the questionnaire and the methodology used to disseminate the questionnaire.

On the other hand, the fact of not having calculated the number of impressions generated for example by each tweet may have underestimated the role of the networks in the dissemination of the questionnaire, and in any case, may have negatively affected our analysis of this dissemination, which would have been considered more extensive if the analysis of the impressions generated by each tweet had been added.

The strengths of the study are also determined by the methodology used for instrument’s distribution and participants’ selection. We have seen how by using social networks, we have managed to bring together nursing professionals from different work environments and from very dispersed geographical locations in a short period of time, in comparison with the traditional way of collecting data.

The use of social networks and online forms to collect information allows us to reduce time and other resources usually devoted to the phases of participant selection, data collection and coding and, therefore, to accelerate the research process and reduce costs.

## Conclusions

Social networks have shown to be a useful tool for research in nursing for its ability to develop networks and disseminate information at high speed and in real time, so it they can also be used as a tool to disseminate instruments of data collection among a given population, also identifiable through these networks.

The results obtained in this study show the usefulness of the Spanish version of the PCQN as an instrument to measure knowledge of nurses in palliative care, and also that Spanish nursing professionals have a limited level of knowledge in the field of palliative care, which would improve through the development of specific training programs in this area.

Social networks have proven to be a useful tool when disseminating data collection instruments and recruiting participants in a research study, improving data collection times and allowing access to a greater number of possible subjects of study. This methods do not affect significantly the results of a study, since similar results to other works, in which the traditional methodology of dissemination of data collection instruments has been used, are obtained.

## References

[1] Cartledge P, Miller M, Phillips B. The use of social-networking sites in medical education. Medical Teacher. 2013 10;35(10):847–57. doi: 10.3109/0142159X.2013.804909 23841681 10.3109/0142159X.2013.804909

[2] Wilcox DT, Godbole PP, Koyle MA. The effect of social media (#SoMe) on journal impact factor and parental awareness in paediatric urology. Journal of pediatric urology. 2017 10;13(5):513.e1–513.e7. doi: 10.1016/j.jpurol.2017.03.027 28483467 10.1016/j.jpurol.2017.03.027

[3] Hütt Herrera H. Las redes sociales: Una nueva herramienta de difusión. Reflexiones. 2012;91:121–8. Available from: https://revistas.ucr.ac.cr/index.php/reflexiones/article/view/1513/1521

[4] Lupton D. ‘Feeling Better Connected’: Academics’ Use of Social Media University of Canberra Canberra News & Media Research Centre; 2014 Available from: https://www.canberra.edu.au/about-uc/faculties/arts-design/attachments2/pdf/n-and-mrc/Feeling-Better-Connected-report-final.pdf

[5] Duke VJA, Anstey A, Carter S, Gosse N, Hutchens KM, Marsh JA. Social media in nurse education: Utilization and E-professionalism. 2017 10;57:8–13. doi: 10.1016/j.nedt.2017.06.009 28683342 10.1016/j.nedt.2017.06.009

[6] Olszewski K, Wolf DM. Follow Me, Like Me, Tweet Me! Implementing Social Media Into Occupational Health. Workplace Health & Safety. 2015 6;63(6):240–4. doi: 10.1177/2165079915580739 26089112 10.1177/2165079915580739

[7] Salzmann-Erikson M. Mental health nurses' use of Twitter for professional purposes during conference participation using #acmhn2016. International Journal of Mental Health Nursing. 2017:1–10. doi: 10.1111/inm.12367 28664648 10.1111/inm.12367

[8] Meng Y, Elkaim L, Wang J, Liu J, Alotaibi NM, Ibrahim GM, et al Social media in epilepsy: A quantitative and qualitative analysis. Epilepsy & behavior. 2017:79–84. doi: 10.1016/j.yebeh.2017.04.033 28554148 10.1016/j.yebeh.2017.04.033

[9] Richardson J, Grose J, Nelmes P, Parra G, Linares M. Tweet if you want to be sustainable: a thematic analysis of a Twitter chat to discuss sustainability in nurse education. Journal of Advanced Nursing. 2016 5;72(5):1086–96. doi: 10.1111/jan.12900 26821875 10.1111/jan.12900

[10] Finfgeld-Connett D. Twitter and Health Science Research. Western Journal of Nursing Research. 2015 10;37(10):1269–83. doi: 10.1177/0193945914565056 25542190 10.1177/0193945914565056

[11] Royal College of Nursing. Positioning nursing in a digital world London: Royal College of Nursing; 2013 Available from: https://www.rcn.org.uk/professional-development/publications/pub-004440

[12] Archibald MM, Clark AM. Twitter and nursing research: how diffusion of innovation theory can help uptake. Journal of Advanced Nursing. 2014 3;70(3):e3–5. doi: 10.1111/jan.12343 24450876 10.1111/jan.12343

[13] Carlquist E, Lee NE, Shalin SC, Goodman M, Gardner JM. Dermatopathology and Social Media: A Survey of 131 Medical Professionals From 29 Countries. Archives of Patholology and Laboratory Medicine. 2018 2;142(2):184–190. doi: 10.5858/arpa.2017-0064-OA 28657771 10.5858/arpa.2017-0064-OA

[14] Huby K, Smith J. Relevance of social media to nurses and healthcare:‘to tweet or not to tweet’. Evidence-based nursing. 2016;19:105–6. doi: 10.1136/eb-2016-102476 27590166 10.1136/eb-2016-102476

[15] Urueña A, Ferrari A, Blanco D, Valdecasa E. Las Redes Sociales en Internet Madrid: Observatorio nacional de telecomunicaciones; 2011 Availlable from: http://www.osimga.gal/export/sites/osimga/gl/documentos/d/20111201_ontsi_redes_sociais.pdf

[16] Alnemer KA, Alhuzaim WM, Alnemer AA, Alharbi BB, Bawazir A, Barayyan OR, et al Are Health-Related Tweets Evidence Based? Review and Analysis of Health-Related Tweets on Twitter. Journal of Medical Internet Research. 2015 10 29;17(10):e246 doi: 10.2196/jmir.4898 26515535 10.2196/jmir.4898 PMC4642373

[17] Kandadai V, Yang H, Jiang L, Yang CC, Fleisher L, Winston FK. Measuring Health Information Dissemination and Identifying Target Interest Communities on Twitter: Methods Development and Case Study of the @SafetyMD Network. JMIR research protocols. 2016 5 5;5(2):e50 doi: 10.2196/resprot.4203 27151100 10.2196/resprot.4203 PMC4873622

[18] Nwosu AC, Debattista M, Rooney C, Mason S. Social media and palliative medicine: a retrospective 2-year analysis of global Twitter data to evaluate the use of technology to communicate about issues at the end of life. BMJ Supportive & Palliative Care. 2015 6;5(2):207–12. doi: 10.1136/bmjspcare-2014-000701 25183713 10.1136/bmjspcare-2014-000701

[19] Adam LM, Manca DP, Bell RC. Can Facebook Be Used for Research? Experiences Using Facebook to Recruit Pregnant Women for a Randomized Controlled Trial. Journal of Medical Internet Research. 2016 9 21,;18(9):e250 doi: 10.2196/jmir.6404 27655184 10.2196/jmir.6404 PMC5052464

[20] Codorniu N, Bleda M, Alburquerque E, Guanter L, Adell J, García F, et al Cuidados enfermeros en Cuidados Paliativos: Análisis, consensos y retos. Index de Enfermería. 2011;20(1–2):71–5. Available from http://scielo.isciii.es/scielo.php?script=sci_arttext&pid=S1132-12962011000100015&lng=es

[21] de Vlieger M, Gorchs N, Larkin PJ, Porchet F. Palliative nurse education: towards a common language. Palliative Medicine. 2004; 7;18(5):401–3. doi: 10.1191/0269216304pm916ed 15332417 10.1191/0269216304pm916ed

[22] Larkin P. Developing a nurse education network across Europe. International Journal of Palliative Nursing. 2005 8;11(8):420–2. doi: 10.12968/ijpn.2005.11.8.19610 16215517 10.12968/ijpn.2005.11.8.19610

[23] Peden J, Grantham D, Paquin M. Hospice palliative care nursing standards: how do these apply to our practice?. Canada: College and Association of Registered Nurses of Alberta; 2005 Apr. Report No.: 61. 15916231

[24] Radbruch L, Payne S. White Paper on Standards and Norms for Hospice and Palliative Care in Europe: Part 1. European Journal of Palliative Care. 2009;16:278–89. Available from: http://www.eapcnet.eu/LinkClick.aspx?fileticket=f63pXXzVNEY%3D&tabid=735

[25] Radbruch L, Payne S. White Paper on Standards and Norms for Hospice and Palliative Care in Europe: Part 2. European Journal of Palliative Care. 2010;17(6):22–33. Available from: http://www.eapcnet.eu/LinkClick.aspx?fileticket=f63pXXzVNEY%3D&tabid=735

[26] Ferrell BR, Dahlin C, Campbell ML, Paice JA, Malloy P, Virani R. End-of-life Nursing Education Consortium (ELNEC) Training Program: improving palliative care in critical care. Critical care nursing quarterly. 2007 7;30(3):206 doi: 10.1097/01.CNQ.0000278920.37068.e9 17579303 10.1097/01.CNQ.0000278920.37068.e9

[27] Shea J, Grossman S, Wallace M, Lange J. Assessment of advanced practice palliative care nursing competencies in nurse practitioner students: implications for the integration of ELNEC curricular modules. The Journal of nursing education. 2010 4;49(4):183–9. doi: 10.3928/01484834-20090915-05 19954137 10.3928/01484834-20090915-05

[28] Takenouchi S, Sasahara T, Miyashita M, Kawa M, Umeda M, Kuwata M, et al Empowering Nurses Through Translating the End-of-Life Nursing Education Consortium: The End-of-Life Nursing Education Consortium–Japan Core Curriculum Project. Journal of Hospice & Palliative Nursing. 2017;19(6):539–549. doi: 10.1097/NJH.0000000000000385

[29] Codorniu N, Guanter L, Molins A, Utor L. Competencias enfermeras en cuidados paliativos Madrid: Sociedad Española de Cuidados Paliativos (SECPAL); 2013 Available from: http://www.secpal.com/%5CDocumentos%5CBlog%5CMONOGRAFIA%203.pdf

[30] Valles-Fernández P, García-Salvador I. Formación básica en cuidados paliativos: estado actual en las universidades de enfermería españolas. Medicina Paliativa. 2013;20:111–4 doi: 10.1016/j.medipa.2013.03.003

[31] Abudari G, Zahreddine H, Hazeim H, Assi MA, Emara S. Knowledge of and attitudes towards palliative care among multinational nurses in Saudi Arabia. International Journal of Palliative Nursing. 2014 9;20(9):435–41. doi: 10.12968/ijpn.2014.20.9.435 25250548 10.12968/ijpn.2014.20.9.435

[32] Abu-Saad Huijer H, Abboud S, Dimassi H. Palliative care in Lebanon: Knowledge, attitudes and practices of nurses. International Journal of Palliative Nursing. 2009 7;15(7):346–53. doi: 10.12968/ijpn.2009.15.7.43425 19648850 10.12968/ijpn.2009.15.7.43425

[33] Carroll G, Brisson DP, Ross MM, Labbé R. The French version of the palliative care quiz for nursing (PCQN-F): development and evaluation. Journal of palliative care. 2005;21(1):27 15895547

[34] Choi M, Lee J, Kim S, Kim D, Kim H. Nurses’ Knowledge About End-of-Life Care: Where Are We?. Journal of Continuing Education in Nursing. 2012 8;43(8):379–84. doi: 10.3928/00220124-20120615-35 22715872 10.3928/00220124-20120615-35

[35] Ross MM, McDonald B, McGuinness J. The palliative care quiz for nursing (PCQN): the development of an instrument to measure nurses' knowledge of palliative care. Journal of Advanced Nursing. 1996 1;23(1):126–37. 8708208 10.1111/j.1365-2648.1996.tb03106.x

[36] Chover-Sierra E, Martínez-Sabater A, Lapeña-Moñux YR. An instrument to measure nurses' knowledge in palliative care: Validation of the Spanish version of Palliative Care Quiz for Nurses. PLoS One. 2017 5 18;12(5):e0177000 doi: 10.1371/journal.pone.0177000 28545037 10.1371/journal.pone.0177000 PMC5436641

[37] Chover-Sierra E, Martínez-Sabater A, Lapeña-Moñux YR. Knowledge in palliative care of nursing professionals at a Spanish hospital. Revista Latinoamericana de Enfermagem. 2017; 25: e2847 doi: 10.1590/1518-8345.1610.2847 29069265 10.1590/1518-8345.1610.2847 PMC5656333

[38] Mayol J, Dziakova J. Value of social media in advancing surgical research. British Journal of Surgery. 2017 12;104(13):1753–5. doi: 10.1002/bjs.10767 29144003 10.1002/bjs.10767

[39] Sinnenberg L, DiSilvestro CL, Mancheno C, Dailey K, Tufts C, Buttenheim AM, et al Twitter as a Potential Data Source for Cardiovascular Disease Research. JAMA Cardiology. 2016 12 1;1(9):1032–1036. doi: 10.1001/jamacardio.2016.3029 27680322 10.1001/jamacardio.2016.3029 PMC5177459

[40] Nash EL, Gilroy D, Srikusalanukul W, Abhayaratna WP, Stanton T, Mitchell G, et al Facebook advertising for participant recruitment into a blood pressure clinical trial. Journal of Hypertension. 2017 12;35(12):2527–2531. doi: 10.1097/HJH.0000000000001477 28704263 10.1097/HJH.0000000000001477

[41] Knapp CA, Madden V, Wang H, Kassing K, Curtis C, Sloyer P, et al Paediatric nurses’ knowledge of palliative care in Florida: A quantitative study. International Journal of Palliative Nursing. 2009 9;15(9):432–9. doi: 10.12968/ijpn.2009.15.9.44255 19957453 10.12968/ijpn.2009.15.9.44255

[42] Autor SH, Storey SL, Ziemba-Davis M. Knowledge of Palliative Care. An Evaluation of Oncology, Intensive Care, and Heart Failure Nurses. Journal of Hospice & Palliative Nursing. 2013;15(5):307–15. doi: 10.1097/NJH.0b013e3182930800

[43] Brazil K, Kaasalainen S, McAiney C, Brink P, Kelly ML. Knowledge and perceived competence among nurses caring for the dying in long-term care homes. International Journal of Palliative Nursing. 2012 2;18(2):77–83. doi: 10.12968/ijpn.2012.18.2.77 22399045 10.12968/ijpn.2012.18.2.77

[44] Kim BH, Kim HS, Yu SJ, Choi S, Jung Y, Kwon S. Evaluation of End-of-Life Nursing Education Consortium-Geriatric Train-the-Trainer Program in Korea. Korean Journal of Adult Nursing. 2012 8;24(4):390–7. doi: 10.7475/kjan.2012.24.4.390

[45] Kim HS, Kim BH, Yu SJ, Kim S, Park SH, Choi S, et al The Effect of an End-of-Life Nursing Education Consortium Course on Nurses' Knowledge of Hospice and Palliative Care in Korea. Journal of Hospice & Palliative Nursing. 2011 7;13(4):222–9. doi: 10.1097/NJH.0b013e318210fdec

[46] Mitchell G, McGreevy J, Preshaw DH, Agnelli J, Diamond M. Care home managers' knowledge of palliative care: a Northern Irish study. International Journal of Palliative Nursing. 2016 5;22(5):230–5. doi: 10.12968/ijpn.2016.22.5.230 27233010 10.12968/ijpn.2016.22.5.230

[47] Raudonis BM, Kyba FCN, Kinsey TA. Long-term care nurses' knowledge of end-of-life care. Geriatric Nursing. 2002;23(6):296–301. 12494000 10.1067/mgn.2002.130270

[48] Wilson O, Avalos G, Dowling M. Knowledge of palliative care and attitudes towards nursing the dying patient. British Journal of Nursing. 2016 6;25(11):600–5. doi: 10.12968/bjon.2016.25.11.600 27281593 10.12968/bjon.2016.25.11.600

[49] Kassa H, Murugan R, Zewdu F, Hailu M, Woldeyohannes D. Assessment of knowledge, attitude and practice and associated factors towards palliative care among nurses working in selected hospitals, Addis Ababa, Ethiopia. BMC palliative care. 2014 3 4;13(1):6 doi: 10.1186/1472-684X-13-6 24593779 10.1186/1472-684X-13-6 PMC3975866

[50] Al Qadire M. Nurses’ Knowledge About Palliative Care. A cross-sectional survey. Journal of Hospice & Palliative Nursing. 2014 2;16(1):23–30. doi: 10.1097/NJH.0000000000000017

[51] Ronaldson S, Hayes L, Carey M, Aggar C. A study of nurses' knowledge of a palliative approach in residential aged care facilities. International journal of older people nursing. 2008 12;3(4):258–67. doi: 10.1111/j.1748-3743.2008.00136.x 20925863 10.1111/j.1748-3743.2008.00136.x

